# Searching for Bacteria in Neural Tissue From Amyotrophic Lateral Sclerosis

**DOI:** 10.3389/fnins.2019.00171

**Published:** 2019-02-26

**Authors:** Ruth Alonso, Diana Pisa, Luis Carrasco

**Affiliations:** Centro de Biología Molecular “Severo Ochoa” (CSIC-UAM), Universidad Autónoma de Madrid, Madrid, Spain

**Keywords:** neurodegenerative disease, amyotrophic lateral sclerosis, polymicrobial infection, next generation sequencing, repeat expansion C9orf72

## Abstract

Despite great efforts in the investigation, the exact etiology of amyotrophic lateral sclerosis (ALS) is a matter of intensive research. We recently advanced the idea that ALS might be caused by fungal infection. Indeed, fungal yeast and hyphal structures can be directly visualized in neural tissue of ALS patients, and a number of fungal species have been identified in the central nervous system (CNS). In the present work, we tested the possibility that bacterial infections can accompany these mycoses. Our findings establish the presence of bacterial DNA in different regions of the CNS from all ALS patients examined. Specifically, we used PCR and next generation sequencing (NGS) to precisely determine the bacterial species present in ALS tissue. Consistent with these findings, immunohistochemistry analysis of CNS sections using specific anti-bacterial antibodies identified prokaryotic cells in neural tissue. Finally, we assayed for the repeat expansion of the hexanucleotide repeat GGGGCC in C9orf72, which is considered the most common genetic cause of ALS in patients, using DNA extracted from ALS CNS tissue. We failed to find this repeated sequence in any of the eleven patients analyzed. Our results indicate that bacterial DNA and prokaryotic cells are present in CNS tissue, leading to the concept that both fungal and bacterial infections coexist in patients with ALS. These observations lay the groundwork for the use of appropriate therapies to eradicate the polymicrobial infections in ALS.

## Introduction

Amyotrophic lateral sclerosis (ALS) is the most common of the motor neuron diseases and is characterized by progressive muscular dystrophy and degeneration of motor neurons, ultimately leading to fatal paralysis in adulthood (Saberi et al., 2015; Huynh et al., 2016; Oskarsson et al., 2018). Whereas most cases of ALS are classified as sporadic, about 5% are familial forms and have a genetic basis (Iguchi et al., 2013). Typically, ALS has a focal presentation with a progressive and selective loss of both cortical (upper) and spinal (lower) motor neurons across multiple body regions (Ravits and La Spada, 2009; Turner et al., 2013). ALS has been related to other neurodegenerative diseases such as frontotemporal dementia (FTD), and the boundaries between these disorders are not clear. A number of modifications in neurons of the frontal cortex, temporal cortex, hippocampus, cerebellum and striatum have been identified in ALS (Saberi et al., 2015) and, as a consequence, cognitive and behavioral impairment are recognized in 30% of cases and 14% also have FTD (Turner et al., 2013).

Despite extensive research, the etiology of ALS remains uncertain. Two major avenues of research have been followed in an attempt to uncover the origin of ALS: characterization of the cytoplasmic granules found in some motor neurons, and identification of genetic mutations in patients with ALS. As occurs in many neurodegenerative syndromes, several proteins have been found to form cytoplasmic aggregates in affected brain regions in the majority of patients. This is exemplified by the presence of ubiquitinated inclusions, some of them containing transactive response-DNA binding protein 43 (TDP-43), and others occasionally containing the protein fused in sarcoma (FUS) (Saberi et al., 2015; Huynh et al., 2016). These proteins are predominantly nuclear, shuttling between the nucleus and cytoplasm (Wang et al., 2004; Lagier-Tourenne et al., 2010) and, under abnormal conditions, they accumulate in the cytoplasm forming a component of stress granules (Fan and Leung, 2016). Besides TDP-43 and FUS, many additional proteins are under investigation as potential participants in ALS pathogenesis, including heterogeneous nuclear ribonucleoprotein A1 (hnRNPA1), hnRNPA2B1, hnRNPA3, TAF15, and EWSR1 (Neumann et al., 2011; Ugras and Shorter, 2012; Thomsen et al., 2013; Le Ber et al., 2014).

A number of mutations in over 25 genes have been described thus far in familial ALS (Corcia et al., 2017), confirming that the genetic background may predispose to ALS and pointing to the participation of a range of genes in the pathology of the disease. Mutations in the gene encoding Cu/Zn-superoxide dismutase (SOD1) were the first to be linked to ALS in patients and remain the most prevalent. Later studies uncovered many others, such as *FUS*, *TARDBP*, and *TBK1* (Leblond et al., 2014; Renton et al., 2014; Tan et al., 2017). More recently, a large hexanucleotide (GGGGCC) repeat expansion in the first intron of the C9orf72 gene (Renton et al., 2014; Herrmann and Parlato, 2018) was identified to account for 35% of familial ALS patients and for ∼5–7% of sporadic cases of European ancestry, whereas it was relatively absent in Asian ALS patients (Majounie et al., 2012; Woollacott and Mead, 2014; Muller et al., 2018). The C9orf72 gene contains twelve exons, with three transcription variants that synthesize two protein isoforms, termed a and b (De Jesus-Hernandez et al., 2011; Renton et al., 2011). The encoded protein is a Rab guanine exchange factor involved in membrane trafficking and autophagy (Levine et al., 2013; Sellier et al., 2016; Webster et al., 2016). Three different mechanisms have been suggested to account for the neuropathology linked to this repeated expansion. One mechanism is the down regulation of C9orf72 gene expression (De Jesus-Hernandez et al., 2011) and the second entails a gain-of-function by sequestration of essential RNA-binding proteins (RBPs) into intranuclear RNA foci *via* their interaction with the tandem repeat expansion in the mRNA (Gendron et al., 2014; Ciesiolka et al., 2017). Indeed, a variety of RBPs can interact with the repeated expansion, particularly proteins belonging to the hnRNP family (Kumar and Ghosh, 2017). A third mechanism involves the formation of aberrant spliced mRNAs bearing the repeat expansion, which can lead to the synthesis of proteins containing dipeptide repeats (DPRs) (Gendron et al., 2013; Tabet et al., 2018). Translation of both sense and anti-sense aberrant C9orf72 mRNAs has been proposed, beginning translation at a CUG codon. This repeat-associated non-AUG translation may lead to the synthesis of a variety of proteins bearing different DPRs, which could associate to form granules involved in cytotoxicity (Kumar and Ghosh, 2017). These three mechanisms are not mutually exclusive and may occur simultaneously (Todd and Petrucelli, 2016), however, only a small percentage of mRNAs in which intron retention occurs contain the repeat expansion. Moreover, since this expansion is in the 5′ untranslated region, translation of this expanded repeat should be very inefficient.

Thus, a central idea in ALS research is that mutated proteins forming after an undefined stress aggregate in granules that become pathological for the correct functioning of motor neurons (Iguchi et al., 2013; Saberi et al., 2015; Huynh et al., 2016). The aggregates might be a consequence of the impairment of protein transport between the nucleus and cytoplasm in the case of TDP-43 or of the synthesis of aberrant proteins containing DPRs (Jovicic et al., 2016; Prpar Mihevc et al., 2017).

We have recently advanced the idea that ALS may be caused by fungal infection (Alonso et al., 2015, 2017b). In this respect, the different mutated genes described in ALS may reflect a genetic susceptibility for infection. Several lines of investigation support the concept that microbial infection occurs in the CNS of ALS patients. The most direct evidence is the demonstration of fungal yeast and hyphal structures in neural tissue from ALS patients (Alonso et al., 2017b), and the identification of different fungal species in different CNS regions by next generation sequencing (NGS). Another line of evidence in support of the infectious etiology of ALS comes from the finding that amyloid peptide exhibits strong antifungal and antibacterial activity and is involved in the innate immune response (Soscia et al., 2010; Kumar et al., 2016). ALS patients contain amyloid plaques in the CNS, as occurs with other neurodegenerative diseases (Schmidt et al., 1998; Farid et al., 2015; Takeda, 2018). Finally, high levels of chitotriosidase (chitinase) have been detected in the cerebrospinal fluid (CSF) of patients with ALS (Varghese et al., 2013a; Pagliardini et al., 2015). This enzyme is synthesized in response to its substrate, chitin, which is a component of the fungal cell wall. These experimental findings cannot be explained simply by the hypothesis that mutations in some genes are the cause of ALS, but they are compatible with the idea that ALS is provoked by disseminated mycoses.

In the present work, we have examined the possibility that bacterial infections accompany these mycoses. Our results support the existence of polymicrobial infections in the CNS of ALS patients.

## Materials and Methods

### Description of ALS Patients

CNS sections and frozen tissue were obtained from 11 patients diagnosed with ALS. The age, gender and code number of each patient are indicated (Supplementary Table 1). All ALS cases studied In this work were sporadic. In addition to ALS, patient 1 was diagnosed with FTD and patient 8 was diagnosed with hippocampal sclerosis. All samples were supplied by the Banco de Tejidos CIEN, Madrid, brain bank and were analyzed anonymously. Sample transfer was carried out according to national regulations concerning research on human biological samples and written informed consent was obtained in all cases. The ethics committee of the Universidad Autónoma de Madrid approved the study. All samples were processed according to a common postmortem protocol followed by Banco de Tejidos CIEN. To avoid contamination, the frozen tissue was handled with sterile instruments in a laminar flow hood.

### DNA Extraction From CNS Tissue and Nested PCR

DNA was extracted from frozen samples as described (Alonso et al., 2017a). To assay for bacterial DNA, we used nested PCR with several primer pairs as described (Alonso et al., 2017a), which amplify a region between the V3–V4 variable region of the prokaryotic 16S rRNA gene. In the first PCR, 4 μl of DNA was denatured at 95°C for 5 min, followed by 35 cycles of 1 min at 94°C, 1 min at 56°C and 3 min at 72°C, using primers 27F and 1492R. The second PCR was performed using 2 μl of the product obtained in the first PCR with forward V3 and reverse V4 internal primers, for 30 cycles of 1 min at 94°C, 1 min at 55°C and 3 min at 72°C (Supplementary Figure 1).

**Figure 1:**
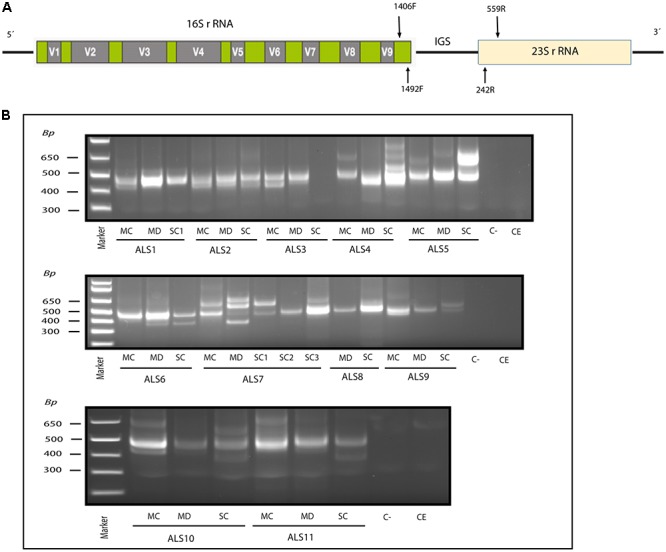
Nested PCR analysis of bacterial DNA extracted from patients with ALS. PCR analysis was carried out as described in section Materials and Methods. **(A)** Schematic representation of bacterial rRNA genes (16S and 23S) and the intergenic sequence (IGS) region, including the location of the primers employed for the different reactions. **(B)** Agarose gel electrophoresis of the DNA fragments amplified by nested PCR; analysis of three CNS regions (MC, MD, and SC) from 11 patients amplifying the IGS region Primers 1406(F) – 559(R) and 1492 (F) –242 (R) were used in the first and second round PCR, respectively, C-, control PCR without DNA; CE, control of DNA extraction without DNA; MC, motor cortex; MD, medulla; SC, spinal cord; SC1, SC2, and SC3, three samples from different regions of the spinal cord.

In addition, the intergenic spacer (IGS) region between rRNA genes of the prokaryotic genome was amplified using the primers 1406(F)/559(R) and 1492(F)/242(R) in the first and second round PCR, respectively (see scheme in Figure 1A). The first PCR was carried out with 4 μl of DNA denatured at 95°C for 5 min, followed by 45 cycles of 1 min at 94°C, 1 min at 55°C and 3 min at 72°C. The second PCR was performed using 2 μl of the product obtained in the first PCR, for 35 cycles of 1 min at 94°C, 1 min at 57°C and 3 min at 72°C. Some PCR products were sequenced by Macrogen Inc. (Seoul, Korea). The sequences have been submitted to the European Nucleotide Archive with the access numbers LR031262–LR031290.

To analyze the hexanucleotide expansion repeat in the C9orf72 gene in the different samples, we performed PCR with 4 μl of DNA denatured at 95°C for 5 min, followed by 45 cycles of 1 min at 94°C, 1 min at 56°C and 3 min at 72°C. The primers used were (F): 5′AGTCGCTAGAGGCGAAAGC3′ and (R): 5′TACGCATCCCAGTTTGAGACG CGGGGGCCGGGGCCGGGGCCGGGG 3′.

### Next Generation Sequencing

NGS was performed as described (Alonso et al., 2017a), and primers were designed to amplify the region between the variable V3–V4 16S rDNA gene. Primers were joined to linker sequences in a first round of PCR (specific product of ∼400 nucleotides). A second PCR was performed on the products using fusion primers containing Illumina and linker sequences. PCR products were sequenced on a MiSeq sequencing platform (Illumina). PCR and sequencing were carried out by the Genomics Unit at the Scientific Park of Madrid. Quality analyses were carried out over reads using FastQC software^1^. All sequences have been submitted to European Genome-phenome Archive with the accession numbers EGAR00001825350–EGAR00001825307.

### Computational Analysis

#### Qiime Analysis

We used QIIME software for metagenomic analysis of bacteria (Caporaso et al., 2010), which is an open-source bioinformatics pipeline for microbiome analysis from raw DNA sequencing data. QIIME is designed to take users from raw sequencing data to publication-quality graphics and statistics. This includes demultiplexing and quality filtering, operational taxonomic unit (OTU) picking, taxonomic assignment and phylogenetic reconstruction, and diversity analyses and visualizations. The adapters from the sequences were deleted using Cutadapt and all sequences with a length shorter than 35 base pairs were discarded. Once sequence set-up was ready, we performed a metagenomic-type analysis that consisted of several steps.

#### Sequence Clustering

The sequences were grouped to define OTUs as reported (Alonso et al., 2018) with a percentage identity of 94%.

#### Principal Component Analysis

Principal component analysis (PCA) was carried out as reported (Alonso et al., 2018).

#### Statistical Analysis

Statistical analysis was performed using the nonparametric Wilconxon–Mann–Whitney test to calculate pairwise comparisons between group levels with corrections for multiple testing. Statistical analyses of species and genera was performed using the Statistical Analysis of Metagenomic Profiles (STAMP) software package (Parks et al., 2014).

#### Immunohistochemistry Analysis

The antifungal antibodies employed have been described previously (Pisa et al., 2015, 2016a). The following bacterial antibodies were used: rabbit polyclonal antibody against *Chlamydophila pneumoniae*, which immunoreacts with the major outer porin (Biorbyt, Cambridge, United Kingdom), used at 1:20 dilution; and mouse monoclonal antibody against peptidoglycan (Thermo Fisher Scientific, Waltham, MA, United States), used at 1:20 dilution. Standard techniques were used for paraffin embedding and sectioning of CNS tissue. Protocols for immunohistochemical analysis have been described (Pisa et al., 2016b). Most of the images were obtained with a Zeiss LSM710 multiphoton confocal laser scanning microscope equipped with the upright AxioImager.M2 stand (Zeiss), running ZEN 2010 software. Images were deconvoluted using Huygens software (4.2.2 p0) and visualized with ImageJ (NIH).

## Results

### Detection of Bacterial DNA in CNS Tissue by Nested PCR

Recent evidence from several laboratories has revealed that bacteria can be detected in brain tissue from ostensibly healthy subjects, albeit at low levels (Branton et al., 2016; Emery et al., 2017; Alonso et al., 2018). Moreover, we and others have recently identified bacteria in the CNS of patients diagnosed with multiple sclerosis (MS) and Alzheimer’s disease (AD) (Branton et al., 2016; Emery et al., 2017; Pisa et al., 2017; Alonso et al., 2018). To test whether bacteria are present in the CNS tissue from patients with ALS, we analyzed DNA isolated from frozen postmortem tissue of the following CNS regions from 11 patients: motor cortex (MC), median bulb (medulla, MD) and spinal cord (SC). Three different regions of the SC are indicated as SC1, SC2, and SC3. As the bulk of this DNA is human, we used nested PCR to enhance the detection of bacterial DNA using primers directed to the IGS region between ribosomal RNA (rRNA) genes (see schematic in Figure 1A). As negative controls, we performed PCR without DNA and without extraction. Results showed that several DNA fragments were amplified in virtually all regions from the 11 patients, but not in the negative controls (Figure 1B). We next extracted and sequenced the individual DNA bands to identify the bacterial species. The most representative species was *Cutibacterium acnes* (formerly known as *Propionibacterium acnes*) (band size 402–568 bp). This bacterium has been reported in previous studies in human CNS (Emery et al., 2017; Alonso et al., 2018). Other species detected included *Corynebacterium sp* (patient ALS5, SC) (band size 630 bp), *Fusobacterium nucleatum* (ALS11, SC) (band size 460 bp), *Lawsonella clevelandesis* (ALS9, SC) (band size 540 bp) and *Streptococcus thermophilus* (ALS8, MD) (band size 488bp). As a complementary approach, we also performed nested PCR of the V3–V4 region of the 16S rRNA gene, which in this case amplified a, similarly, sized DNA fragment (400 bp) in all patients except patient ALS10 (Supplementary Figure 1). Sequencing of the extracted DNA showed that the amplified product corresponded to *Burkholderia* species (Supplementary Table 1). These results show that bacterial species are present in the CNS tissue from patients with ALS, and some species are preferentially amplified depending on the primers employed. All dises sequences are publically available at ENA.

### Detection of Bacterial DNA From ALS Patients by NGS

**Figure 2:**
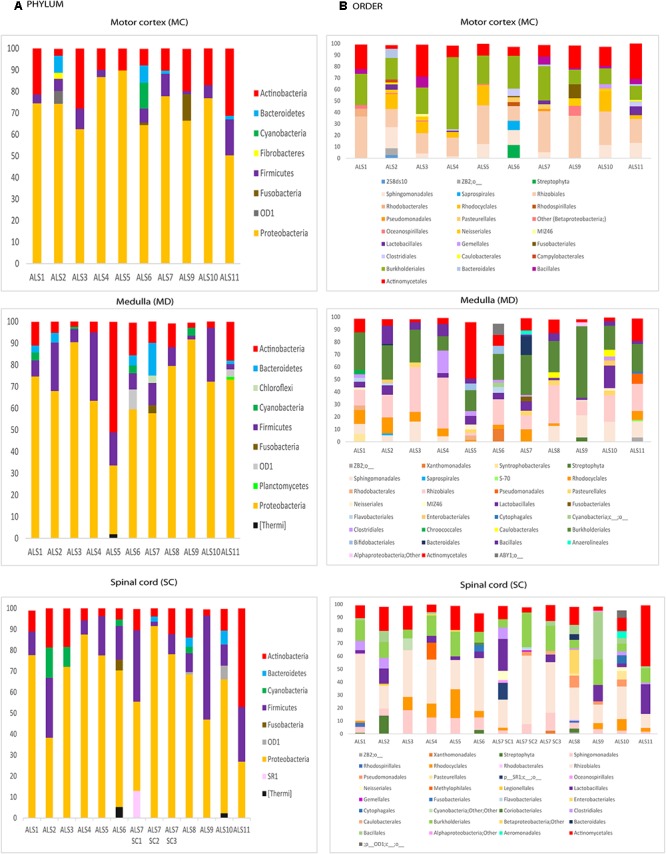
Distribution of bacteria phyla and orders obtained by NGS of DNA from eleven ALS patients. Computational analyses of the sequences obtained on the Illumina platform using Qiime classified the data into bacteria phyla and orders. **(A)** Panels show the results of bacteria phyla obtained from three CNS regions. **(B)** Panels show the results of bacteria orders obtained from three CNS regions. SC1, SC2 and SC3, three samples from different regions of the spinal cord.

We next used NGS to comprehensively analyze the DNA samples from the different CNS regions of the 11 patients. Accordingly, an internal region of the 16S rRNA gene was amplified and sequenced on the Illumina Platform. The number of sequences obtained for each sample varied from 140,818 to 238,088. The bacterial species detected are listed in Supplementary Table 2, with only those with a presentation > 1% shown. In concordance with previous reports, a great variety of species were identified using this technique. The order *Sphyngomonodales*, the family *Methilobacteriaceae* and the genus *Cupriavidus* seemed to be the most prevalent bacterial groups in ALS patients. Interestingly, *Sphyngomonodales* has not previously been reported in the CNS of control subjects (Branton et al., 2016; Emery et al., 2017; Alonso et al., 2018). The bacterial phyla and orders from the three CNS regions (MC, MD and SC) of the ALS patients are shown in Figure 2. The phyla *Actinobacteria and Proteobacteria* were found in all three regions of the 11 ALS patients analyzed. The phyla *Firmicutes* and *Bacteriodetes* were also prevalent, whereas other phyla were less represented. For example, *Fusobacteria* was found in only one patient, in all three CNS regions. In addition, we detected a great variability in the bacterial orders present in ALS patients. For instance, *Actinomycetales, Burkholderiales* and *Rhizobiales* were detected in all ALS patients, whereas *Xanthomonadales* was found only in two patients (ALS6, MD and ALS7, SC3). These findings reveal the irregular range of bacteria in the different samples tested, indicating that these bacteria are likely not due to contamination during DNA extraction and NGS analysis.

### Principal Component Analysis

**Figure 3:**
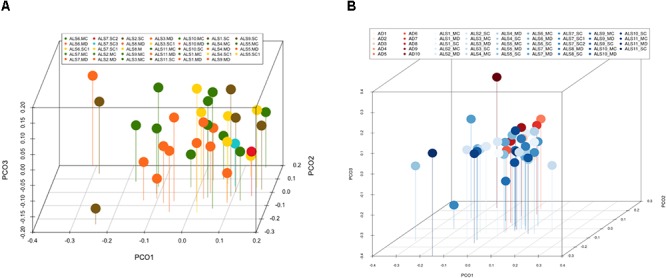
Principal component analysis of bacteria of CNS regions from ALS patients and AD patients. **(A)** Three-dimensional (3D) principal component analysis (PCA) between different CNS regions from 11 ALS patients. **(B)** 3D PCA between ALS and AD patients. MC, motor cortex; MD, medulla; SC, spinal cord, SC1, SC2, and SC3, three samples from different regions of the spinal cord.

Multivariate PCA of the results obtained from the three CNS regions in ALS patients is shown in Figure 3A. The distribution of bacterial species was similar between the three regions, and statistical analysis using the Wilconxon–Mann–Whitney test showed that there were no significant differences between each CNS region (*p* > 0.05).

**Figure 4:**
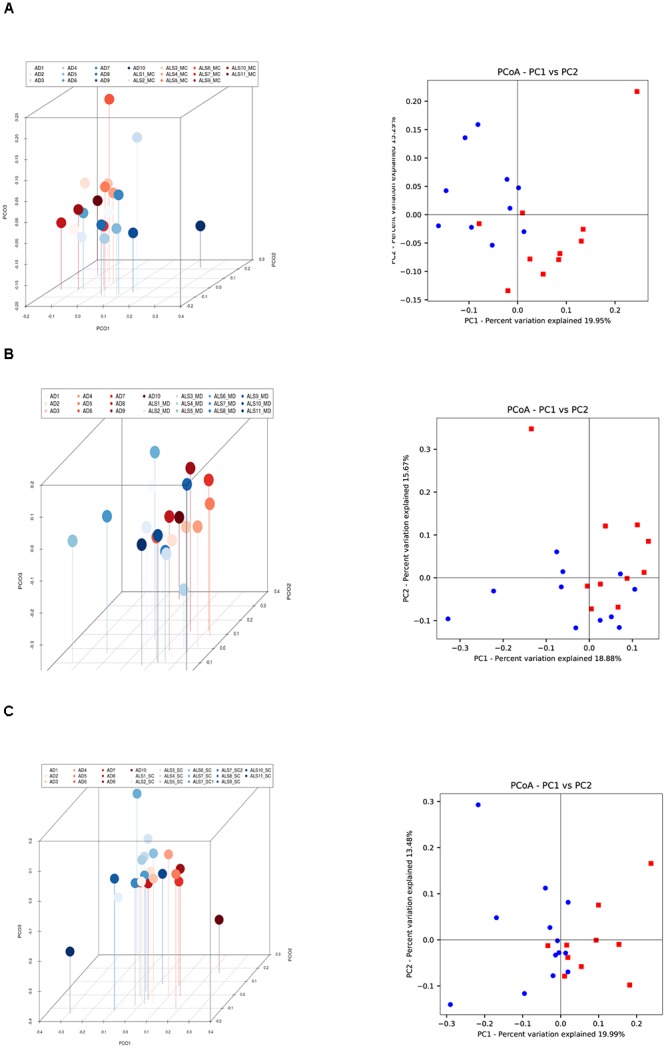
Three- and two-dimensional principal component analysis of bacteria of CNS regions from ALS patients and AD patients. Comparison of three separate CNS regions between AD and ALS patients. **(A)** Left panel: three-dimensional (3D) principal component analysis (PCA) between MC regions of ten ALS patients (plots in red) and 10 AD patients (plots in blue). Right panel: 2D PCA. **(B)** Left panel: 3D PCA between MD regions of eleven ALS patients (plots in blue) and ten AD patients (plots in red). Right panel: 2D PCA. **(C)** Left panel: 3D PCA between SC regions of 11 ALS patients (plots in blue) and 10 AD patients (plots in red). Right panel: 2D PCA. The UniFrac method was used to calculate this parameter. MC, motor cortex; MD, medulla; SC, spinal cord.

The PCA of the present NGS data set was next compared with that previously reported for patients with AD and control subjects (Alonso et al., 2018). The distribution between ALS and AD patients was similar (Figure 3B), however, when the comparison was made between each region from ALS patients and the enthorrinal region from AD patients, the distribution was significantly different (Figure 4A–C). These findings were supported by White’s nonparametric *t*-test with *p*-values adjusted for multiple testing using the Benjamini–Hochberg approach in STAMP (Supplementary Figure 2), showing the genera- and species-dependent significant differences. For instance, the bacterial genera *Acrobacter, Thermomonas, Hemophylus, Propionibacterium, and Corynebacterium* are more represented in MC, MD, and SC regions in ALS patients than in AD patients. Further, *Propionibacterium acnes* is the most representative in each region with respect to AD patients.

**Figure 5:**
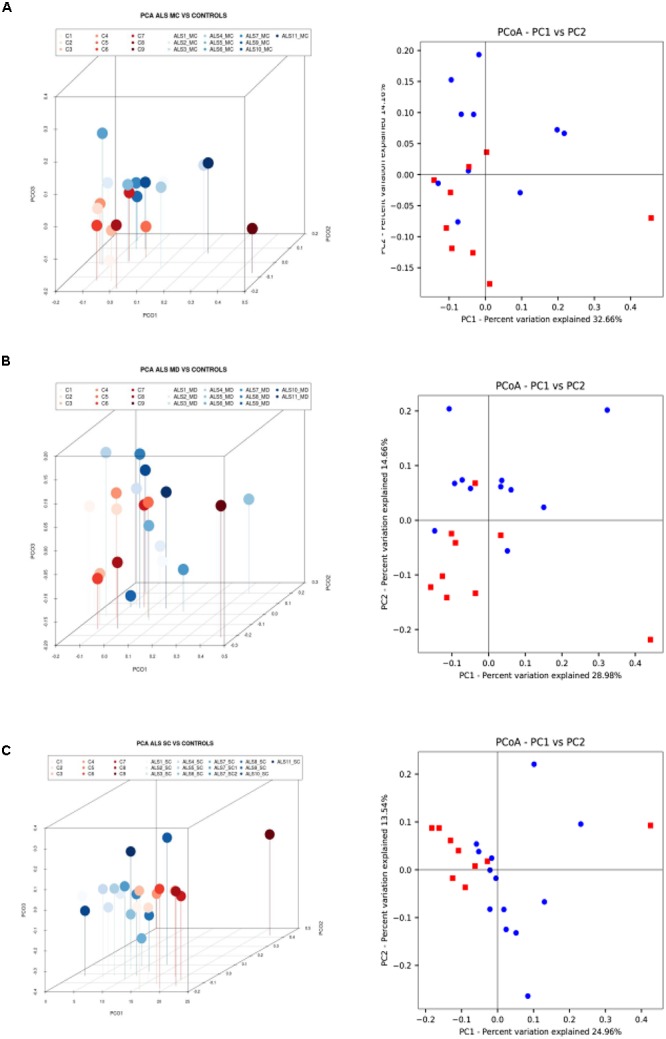
Three- and two-dimensional principal component analysis of bacteria of CNS regions from ALS patients and control samples. **(A)** Left panel: three-dimensional (3D) principal component analysis (PCA) between MC regions of 10 ALS patients (plots in blue) and 9 control samples (plots in red). Right panel: 2D PCA. **(B)** Left panel: 3D principal component analysis between MD regions from 11 ALS patients (plots in blue) and 9 control samples (plots in red). Right panel: 2D PCA. **(C)** Left panel: 3D PCA between SC regions from 11 ALS patients (plots in blue) and 9 control samples (plots in red). Right panel: 2D PCA. The UniFrac method was used to calculate this parameter. MC, motor cortex; MD, medulla; SC, spinal cord.

Finally, we extended this analysis to compare ALS patients with control subjects (Figure 5). Results indicated that there was a small difference in distribution between each region from ALS patients and the control subjects. Only one control (C9) was not in the clustered group. These observations were also supported by the statistical analysis, and we conclude that all ALS samples (MC, MD, and SC) are significantly different from the control group.

### Detection of Bacteria in CNS Tissue by Immunohistochemistry

**Figure 6:**
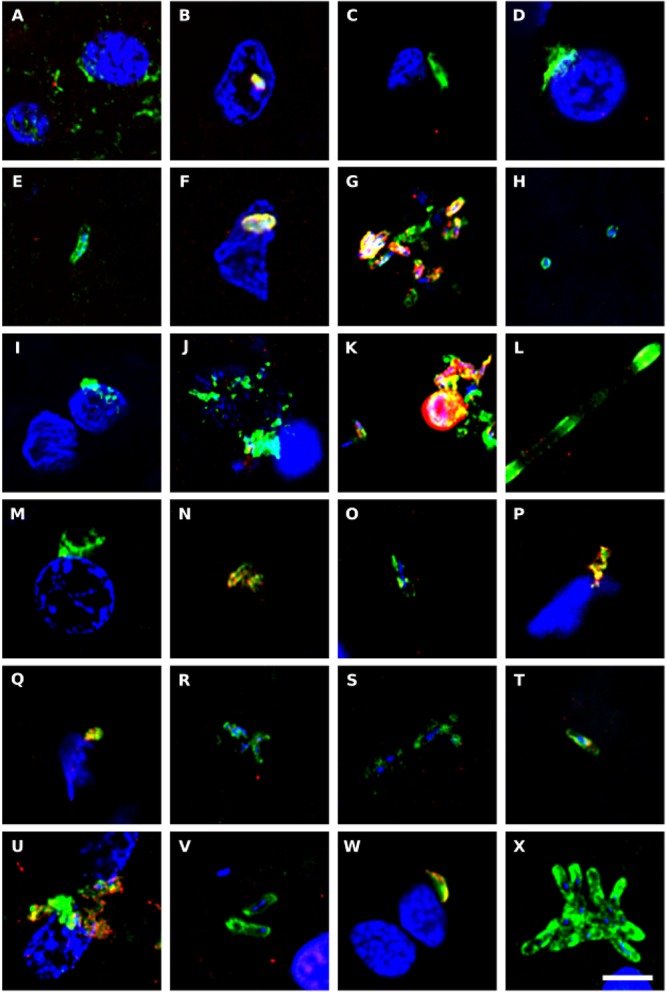
Immunohistochemistry to detect peptidoglycan in brain tissue from ALS patients. Double immunostaining and confocal microscopy were carried out as indicated in section Materials and Methods. CNS sections were immunostained with a mouse monoclonal anti-peptidoglycan antibody (green) (1:20 dilution) and a rabbit polyclonal anti-*C. albicans* antibody (red) (1:500 dilution). DAPI staining of nuclei appears in blue. Scale bar: 5 μm. **(A–C)** ALS2; **(D–F)** ALS3; **(G–I)** ALS4; **(J,K)** ALS5; **(L–N)** ALS6; **(O)** ALS1; **(P)** ALS7; **(Q,R)** ALS8; **(S)** ALS9; **(T–V)** ALS10; and **(W,X)** ALS11. **(A,D,G,J,L,Q,S,T,W)** MC section; **(B,E,H,K,M,O,U,X)** MD section; and **(C,F,I,N,P,R,V)** SC section.

To complement the bacterial DNA study, we performed immunohistochemistry to directly test for prokaryotic structures in the CNS of ALS patients. Initially, we performed double immunofluorescence analysis using two different antibodies: a mouse monoclonal antibody against bacterial peptidoglycan (shown in green), and a rabbit polyclonal antibody against *Candida albicans* (shown in red) (Figure 6). Several prokaryotic morphologies could be detected in different CNS regions by confocal microscopy. For example, a bacilliform structure of 4–7 μm in size was recognized with the anti-peptidoglycan antibody (Figure 6V,X). Smaller bacilli of 1–2 μm were also immunostained with this antibody in some sections (Figure 6B,G). These bacilli are clearly differentiated from yeast-like structures that were recognized with the anti-*C. albicans* antibody (see for instance Figure 6K). In addition to these prokaryotic morphologies, a variety of immunopositive microbial structures were detected, in good agreement with our recent study (Alonso et al., 2017b). Some of these microbial cells were clearly intranuclear (Supplementary Figure 3). Different bacilli-like morphologies that stained with the anti-peptidoglycan antibody could also be clearly seen. The range in sizes of these structures suggests that they represent different species. Moreover, yeast-like cells and some hyphae were also evident. Most likely, the structures immunostained with the anti-peptidoglycan antibody are prokaryotic in origin, although the anti-*C. albicans* antibody not only cross-reacts with several fungal species, but also with some bacteria (Pisa et al., 2017). Nevertheless, none of these antibodies recognizes human cells.

**Figure 7:**
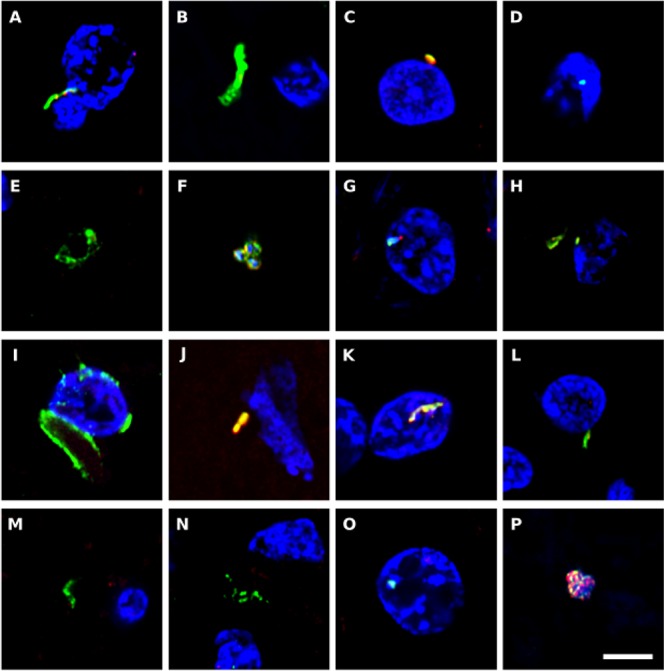
Immunohistochemistry to detect *C. pneumoniae* antigens in brain tissue from ALS patients. Double immunostaining and confocal microscopy were carried out as indicated in section Materials and Methods. CNS sections were immunostained with a rabbit polyclonal *C. pneumoniae* antibody (green) (1:20 dilution) and a rat polyclonal anti-*T. viride* antibody (red) (1:20 dilution). DAPI staining of nuclei appears in blue. Scale bar: 5 μm. **(A,B)** ALS2; **(C,D)** ALS3; **(E)** ALS5; **(F–H)** ALS6; **(I,J)** ALS1; **(K)** ALS7; **(L,M)** ALS8; **(N)** ALS9; **(O)** ALS10; and **(P)** ALS11. **(A,C,F,I,K,L,P)** MC section; **(G,N,O)** MD section; and **(B,D,E,H,J,M)** SC section.

We also immunostained the samples with a rabbit polyclonal antibody against *Chlamydophila pneumoniae* (shown in green) and with a rat polyclonal antibody raised against the fungus *Trichoderma viride* (shown in red) (Figure 7). It must be taken into consideration that these two antibodies can cross-react with prokaryotic and yeast-like cells (Pisa et al., 2017). Both the anti-*C. pneumoniae* antibody and the anti-*T. viride* antibody recognize a variety of bacteria and also immunoreact with several yeast species. However, the spectrum of species recognized by both antibodies is different. Notably, this double immunofluorescence analysis also revealed a number of microbial structures (Figure 7). In some instances, small rounded prokaryotic-like cells were evidenced (see Figure 7C,P). On other occasions, larger rounded cells or even elongated structures were detected (Figure 7A,B,J–M).

In conclusion, a variety of prokaryotic-like structures can be revealed using immunohistochemistry analysis of CNS sections from patients with ALS. These findings are consistent with the results found with PCR and NGS, demonstrating the existence of a variety of bacterial species.

### Detection of Corpora Amylacea by Immunohistochemistry

**Figure 8:**
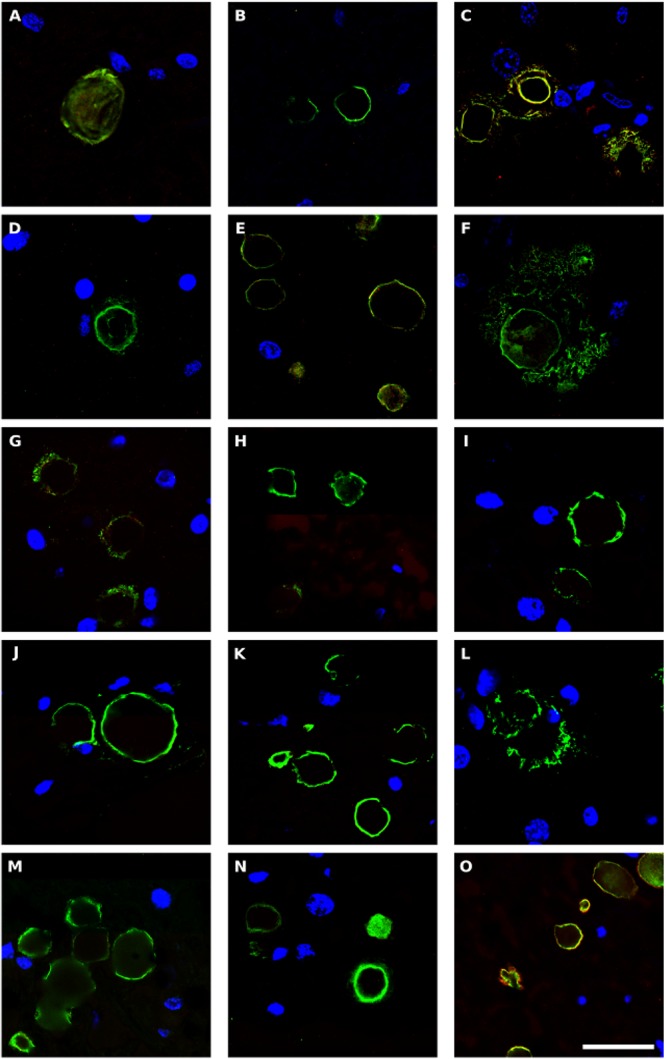
Bacterial proteins in corpora amylacea from ALS patients. Double immunostaining and confocal microscopy were carried out as indicated in section Materials and Methods. CNS sections were immunostained with a mouse monoclonal anti-peptidoglycan antibody (green) (1:20 dilution) and a rabbit polyclonal anti-*C. albicans* antibody (red) (1:500 dilution). DAPI staining of nuclei appears in blue. Scale bar: 25 μm. **(A,B)** ALS2; **(C)** ALS3; **(D,E)**, ALS4; **(F)** ALS5; **(G,H)** ALS7; **(I,J)** ALS8; **(K)** ALS9; **(L–N)** ALS10; and **(O)** ALS11. **(A,D,F,L)** MC section; **(G,I,K,M)** MD section; and **(B,C,E,H,J,N,O)** SC section.

Corpora amylacea (CA) are small (10–50 μm) basophilic bodies that have been identified in several neurodegenerative diseases including AD and Parkinson’s disease (Pisa et al., 2016a). By proteomic analysis, we recently demonstrated that in addition to human proteins, CA purified from brain tissue of patients with AD contain fungal and bacterial proteins (Pisa et al., 2018). We next examined for CA in ALS patients, and whether they stained positive with anti-peptidoglycan or anti-*C. albicans* antibodies. CA were found in several tissue sections from different ALS patients, and a clear immunostaining with anti-peptidoglycan antibodies (green) was observed in many of these CA (Figure 8). In some instances, immunoreactivity of CA with a *C. albicans* antibody (red) was also observed (Figure 8C,E,O). This result is consistent with the concept that CA contain both fungal and bacterial macromolecules and their function might be to collect the cellular and microbial debris provoked by infections in the brain (Pisa et al., 2018). Since CA are generated over long time periods (months or even years), these microbial proteins were likely sequestered in CA when the patients were alive.

### Analysis of the Exanucleotide Expansion Repeat in C9orf72

About 10% of ALS cases contain a repeated expansion in C9orf72, which has also been found in patients with several neuropathological diseases and in control subjects (Woollacott and Mead, 2014). Our next goal was to test for this repeated expansion in the ALS patients studied in this work. We performed PCR analysis in the MC and MD regions of the 11 ALS patients, since somatic variations of this repeat expansion could exist. Results of the amplification and sequencing are listed in Supplementary Table 3. We found three GGGGCC repeats in most patients (ALS1–7 and ALS9), whereas only two repeats were found in two patients (ALS10, MC and ALS11, MC). For each patient the repeat was the same in the two CNS region tested. Thus, the number of repeats is far below that required to cause pathogenicity (above 30 repeats) (Cleary et al., 2016).

## Discussion

Research on the etiology of ALS suggests that this disease has a genetic component. Particularly, the possibility that a hexanucleotide repeat expansion in C9orf72 is responsible for ALS has been put forward (Corcia et al., 2017). The rationale behind this proposal is that the synthesis of aberrantly spliced mRNA retaining intron 1 bearing the repeat expansion can interact and sequester some crucial RBPs (Ciesiolka et al., 2017). In addition, protein synthesis directed by this repeat expansion can lead to the production of aberrant peptides (Ash et al., 2013). Both mechanisms would be detrimental for cell viability. The synthesis of aberrant peptides would induce the formation of peptide aggregates in the cytoplasm and the nucleus leading to cell death (Iguchi et al., 2013; Saberi et al., 2015; Huynh et al., 2016; Kumar and Ghosh, 2017; Tabet et al., 2018). However, this does not apply to the bulk of ALS cases, since this repeat expansion is infrequent in non-Caucasian patients, and is not found in about 90% of ALS patients of European ancestry (Renton et al., 2014). Curiously, the repeat expansion has also been found in a number of patients with other neurological diseases including AD and PD. Moreover, intermediate numbers of the repeat expansion have also been found in healthy control subjects (Woollacott and Mead, 2014). Thus, it could be possible that this repeat expansion predisposes to neurological diseases. In this sense, its toxic effect on cellular physiology might influence the acquisition of microbial infections. In fact, the transcriptome in neural tissue of ALS cases indicates alterations in mRNA transcripts involved in the immune response (Prudencio et al., 2015).

We have advanced the concept that microbial infection contributes to the pathology of ALS, based on the demonstration that fungal proteins and DNA can be detected in the CSF and in the CNS of ALS patients (Alonso et al., 2015). Furthermore, the direct visualization of yeast-like cells and hyphae provides compelling evidence that fungal infection is present in different CNS regions (Alonso et al., 2017b). These fungal structures were present in all ALS patients analyzed and the species present in the CNS were precisely identified by NGS, rendering the genera *Candida, Malassezia, Fusarium, Botrytis*, *Trichoderma*, and *Cryptococcus* as those most prominent in ALS patients. Our present findings indicate that in addition to mycoses, bacterial infection can coexist in neural tissue pointing to the existence of polymicrobial infections in the CNS of these patients. We used both nested PCR and NGS assays to perform an exhaustive analysis of prokaryotic microbes that colonize the CNS of ALS patients, revealing a great variety of bacterial species. Thus, we now have a general idea about the microbiota, both fungal and bacterial, which exists in the CNS of ALS.

The concept that the etiology of ALS is microbial in origin can go some way to explain all of the findings reported for the neuropathology of this disease. First, microbial infection is consistent with neuroinflammation in ALS, with a clear infiltration of T lymphocytes (Henkel et al., 2004; Hooten et al., 2015; Ransohoff, 2016). In fact, mycoses would lead to the infiltration of immune cells into neural tissues, and the decrease in Th2 lymphocytes correlates with the rapid progression of the disease (Henkel et al., 2013). Second, the high levels of chitinase found in the CSF of ALS patients could be due to the presence of fungal chitin, which induces the synthesis of chitinase (Varghese et al., 2013b; Pagliardini et al., 2015). Third, the acquisition of microbial infections is consistent with the sudden appearance of the disease after several decades of life. Moreover, the focal presentation of ALS, followed by its spread to other neighboring CNS areas, can be clearly explained if ALS is caused by polymicrobial infections. Fourth, infection could trigger a stress response in neural cells, blocking the shuttling of proteins between the nucleus and cytoplasm and leading to the formation of stress granules and cell death. Fifth, it is well established that the genetic background of a given individual also affects the susceptibility to microbial infection (Carvalho et al., 2010; Glocker and Grimbacher, 2010; Ok et al., 2011; Maskarinec et al., 2016). Thus, the genetic predisposition for ALS could reflect the susceptibility for microbial colonization. Strengthening this concept, the first mutated gene discovered in familial ALS was *SOD1*, which forms part of the innate immune response and is crucial for host defense against microbes (Moisse and Strong, 2006; Lionakis, 2014). Therefore, to the best of our knowledge, none of the known pathological events of ALS discard the possibility that this disease is caused by polymicrobial infections.

By contrast, none of the other hypotheses put forward to account for the pathology of ALS is sufficiently robust to provide a logical explanation for all of the described observations. In particular, the induction of chitinase and the neuroinflammation with infiltrates of immune cells cannot be explained by defects in protein transport between the nucleus and cytoplasm. Further, since C9orf72 is ubiquitously expressed in human tissues, leading to aberrant proteins from very early in development, why does the toxicity and clinical symptoms of ALS manifest decades after birth?

Another consideration of interest is the presence of amyloid deposits in ALS, as occurs in other neurodegenerative diseases (Schmidt et al., 1998; Farid et al., 2015; Takeda, 2018). Amyloid peptide exhibits strong antifungal and antibacterial activity and forms part of the innate immune response (Soscia et al., 2010; Kumar et al., 2016). Accordingly, microbial infections would stimulate the immune response and trigger the synthesis of amyloid peptide. The fact that there are large differences in the severity of clinical symptoms between patients with ALS, which are reflected in the survival time after diagnosis, is also consistent with the idea that mixed microbial infections – which vary from patient to patient – are responsible for the disease. Moreover, the overlapping clinical symptoms between several neurodegenerative diseases are also in good agreement with the hypothesis that they can be caused by a variety of mixed infections. Indeed, we have provided evidence that both fungal and bacteria colonize different CNS regions in AD (Alonso et al., 2017a, 2018; Pisa et al., 2017). The confirmation that these diseases are caused by fungi or bacteria, or both, should come from clinical trials using already approved antifungal and antibacterial agents. Patients with these neurodegenerative diseases do not have to wait for the development of new therapeutic agents. These studies using safe antimicrobial compounds could be started immediately after approval of these trials.

## Data Availability

The datasets generated for this study can be found in European Genome-phenome Archive, EGAR00001825350–EGAR00001825307.

## Author Contributions

DP performed the immunohistochemistry analyses. RA carried out the PCR and NGS analysis. LC designed the study and wrote the paper. All authors discussed the results and commented on the manuscript.

## Conflict of Interest Statement

The authors declare that the research was conducted in the absence of any commercial or financial relationships that could be construed as a potential conflict of interest.

## Footnotes

1 http://www.bioinformatics.babraham.ac.uk/projects/fastqc/
