# Supplementary material for: Searching for Bacteria in Neural Tissue From Amyotrophic Lateral Sclerosis
Source: Front Neurosci. 2019 Feb 26;13:171. doi: 10.3389/fnins.2019.00171 (PMC6399391; doi:10.3389/fnins.2019.00171)
Supplement: Supplementary file 1 [file Data_Sheet_1.PDF]

SEARCHING FOR BACTERIA IN NEURAL TISSUE

FROM AMYOTROPHIC LATERAL SCLEROSIS

Ruth Alonso, Diana Pisa and Luis Carrasco*

Centro de Biología Molecular "Severo Ochoa" (CSIC-UAM). c/Nicolás Cabrera, 1.

Universidad Autónoma de Madrid. Cantoblanco. 28049 Madrid. Spain.

+RA and DP contributed equally to this work.

*Corresponding author

Email address: lcarrasco@cbm.csic.es. Telephone number: +34 91 497 84 50

Running title: ALS and microbial infection

Supplementary figure 1. **Nested PCR analysis of bacterial DNA extracted from ALS patients.**

PCR analysis was carried out as described in Materials and Methods. Nested PCR analysis of CNS regions from 11 patients amplifying the bacterial 16S rRNA gene. The primers employed were 27F & 1492R for the first round PCR and V3 & V4 for the second PCR. C, control PCR without DNA; CE, control of DNA extraction without DNA; MC, motor cortex; MD, medulla; SC, spinal cord; SC1, SC2 and SC3, three samples from the spinal cord.

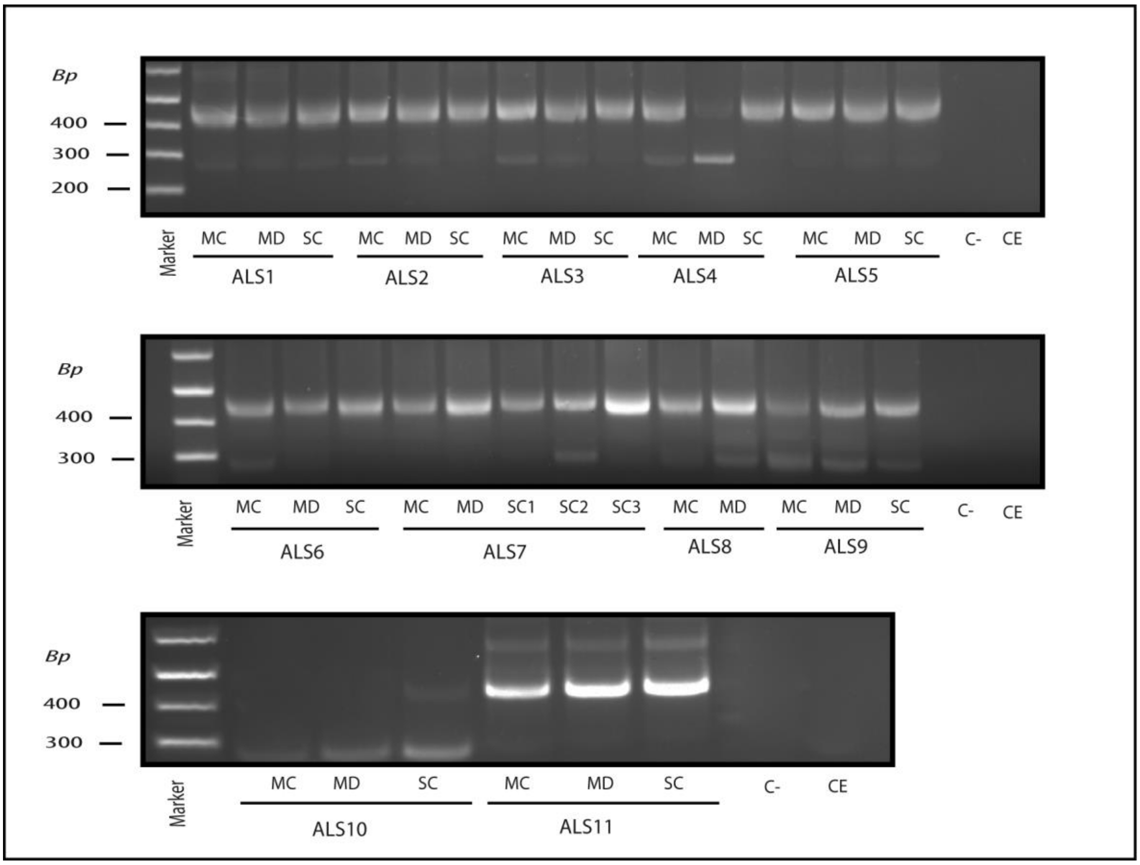

Supplementary figure 2. Genera- and species-dependent statistical analysis between ALS and AD patients.

Statistical analysis was performed using STAMP*. A) Left panel: statistical analysis of genera between the motor cortex region of ALS patients and the enthorrinal region of AD patients. Right panel: statistical analysis of species between the motor cortex region of ALS patients and the enthorrinal region of AD patients. B) Left panel: statistical analysis of genera between the medulla region of ALS patients and the enthorrinal region of AD patients. Right panel: statistical analysis of species between the medulla region of ALS patients and the enthorrinal region of AD patients. C) Left panel: statistical analysis of genera between the spinal cord region of ALS patients and the enthorrinal region of AD patients. Right panel: statistical analysis of species between the spinal cord region of ALS patients and the enthorrinal region of AD patients.

* White's nonparametric t-test with p-values adjusted for multiple testing using the Benjamini-Hochberg approach.

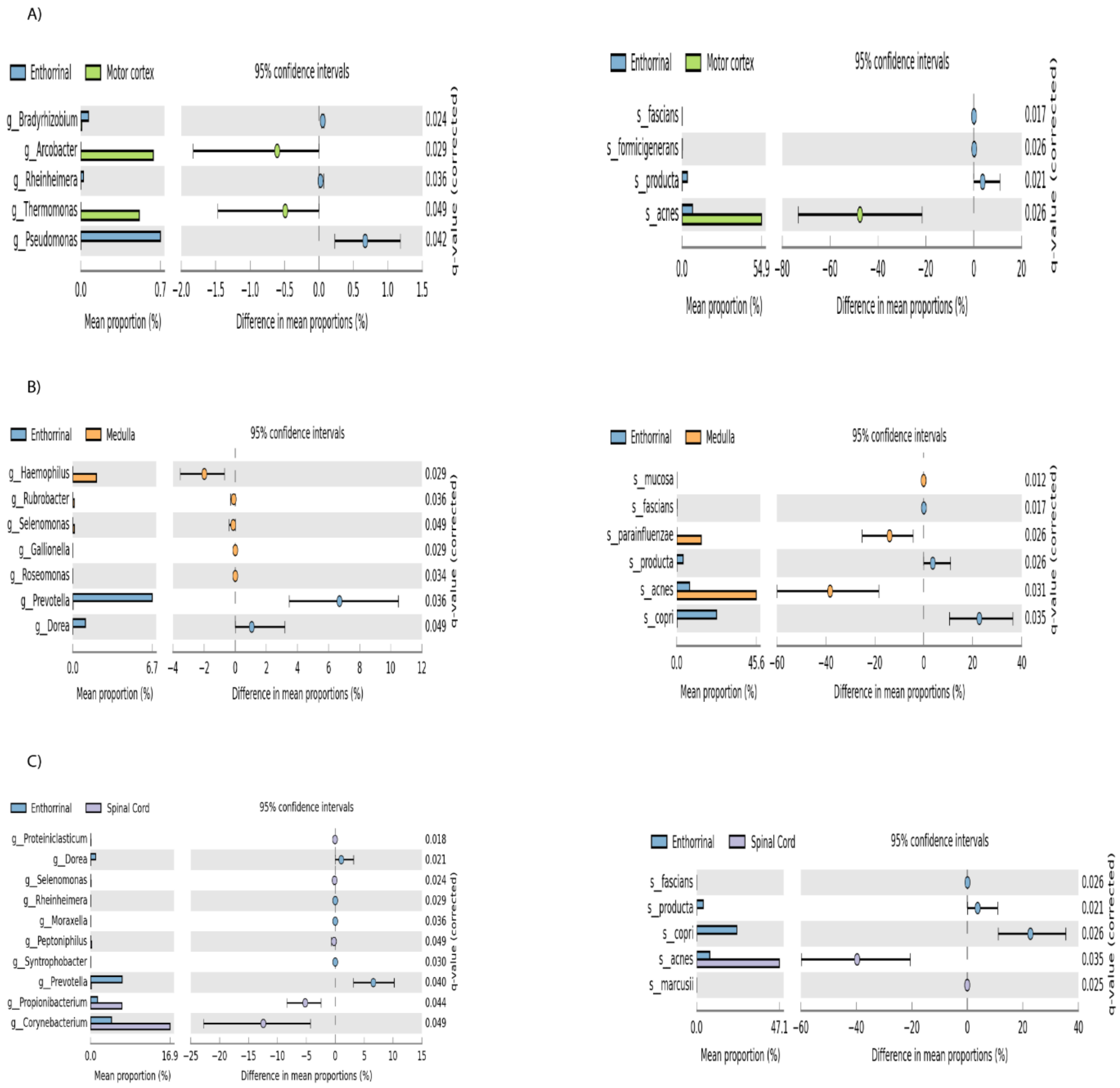

Supplementary figure 3. Immunohistochemistry to detect peptidoglycan in brain tissue from ALS patients

Double immunostaining and confocal microscopy were carried out as indicated in Materials and Methods. CNS sections were immunostained with a mouse monoclonal anti-peptidoglycan antibody (green) (1:20 dilution) and a rabbit polyclonal anti-*C. albicans* antibody (red) (1:500 dilution). DAPI staining of nuclei appears in blue. Scale bar: 5 µm. Panels A–J and P–R, ALS4; panels K and S, ALS5; and panels L–O: ALS11. Panels A–K, P and R, S: motor cortex (MC); and panels L–O and Q: medulla (MD).

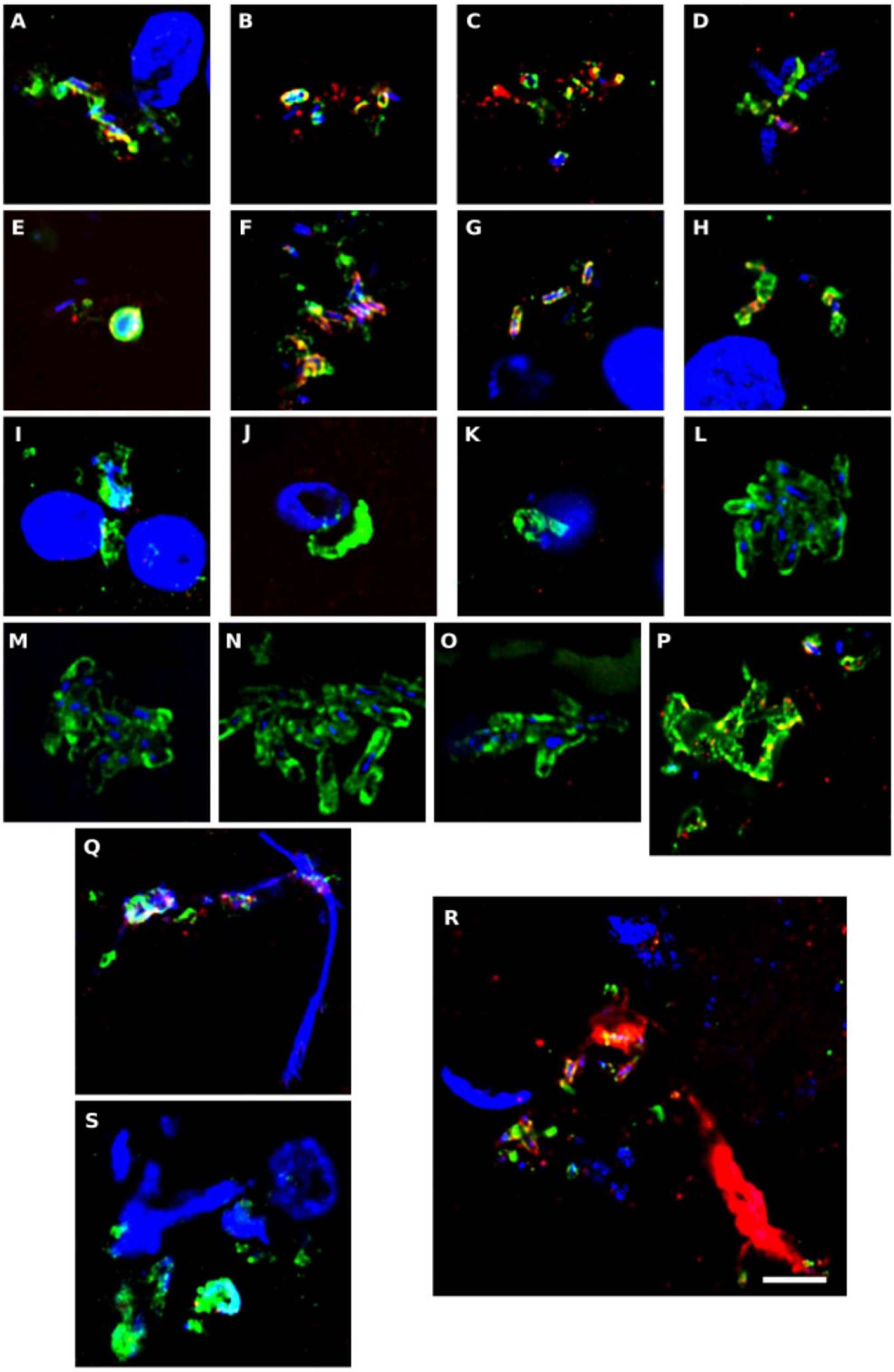
A
B
C
D
E
F
G
H
I
J
K
L
M
N
O
P
Q
R
S
