# Supplementary material for: Searching for Bacteria in Neural Tissue From Amyotrophic Lateral Sclerosis
Source: Front Neurosci. 2019 Feb 26;13:171. doi: 10.3389/fnins.2019.00171 (PMC6399391; doi:10.3389/fnins.2019.00171)
Supplement: Supplementary file 2 [file Data_Sheet_2.PDF]

SEARCHING FOR BACTERIA IN NEURAL TISSUE FROM AMYOTROPHIC LATERAL SCLEROSIS

Ruth Alonso, Diana Pisa and Luis Carrasco*

Centro de Biología Molecular "Severo Ochoa" (CSIC-UAM). c/Nicolás Cabrera, 1. Universidad Autónoma de Madrid. Cantoblanco. 28049 Madrid. Spain.

+RA and DP contributed equally to this work.

*Corresponding author

Email address: lcarrasco@cbm.csic.es. Telephone number: +34 91 497 84 50

Running title: ALS and microbial infection

Supplementary Table I. Bacterial species detected in three regions (MC, MD and SC) from eleven ALS patients using nested PCR and DNA sequencing

Patients	Age	Gender	MC	MD	SC
ALS1	74	Female	*Burkholderia cepacia (422 bp)*	*Burkholderia sp (330 bp)*	*Burkholderia sp (421 bp)*
ALS2	67	Female	*Burkholderia sp (421 bp)*	*Burkholderia sp (421 bp)*	*Burkholderia sp (320 bp)*
ALS3	69	Female	*No blast*	*Burkholderia sp (421 bp)*	*Burkholderia sp (421 bp)*
ALS4	79	Female	*Uncultured burkholderia (422 bp)*	*- No band*	*Uncultured burkholderia (420 bp)*
ALS5	ND	Male	*Uncultured burkholderia (422 bp)*	*Burkholderia sp (423 bp)*	*Burkholderia sp (426 bp)*
ALS6	41	Male	*Burkholderia sp (400 bp)*	*Uncultured burkholderia (420 bp)*	*Uncultured burkholderia (418 bp)*
ALS7	56	Female	*Burkholderia sp (317 bp)*	*Uncultured B protebacterium (415 bp)*	*Uncultured burkholderia (317 bp)*
ALS8	67	Female	*Uncultured bacterium (317 bp)*	*Burkholderia sp (343 bp)*	*Uncultured burkholderia (352 bp)*
ALS9	88	Male	*Uncultured burkholderia (421 bp)*	*Uncultured burkholderia (422 bp)*	*Uncultured B protebacterium (411 bp)*
ALS10	71	Female	*- No band*	*- No band*	*- No band*
ALS11	69	Female	*Burkholderia sp (422 bp)*	*Burkholderia sp (422 bp)*	*Burkholderia sp (416 bp)*

MC: Motor cortex; MD: Medulla; SC: Spinal cordal; ND: no data.

Supplementary Table II. Bacterial species detected in three regions (MC, MD and SC) from eleven ALS patients using NGS

ALS1 MC		ALS1 MD		ALS1 SC		ALS2 MC		ALS2 MD		ALS2 SC	
Number of reads:235361		Number of reads:180951		Number of reads:230744		Number of reads:		Number of reads:167753		Number of reads:140818	
Sequence joined:93,1%		Sequence joined:92,2%		Sequence joined:93,5%		Sequence joined:		Sequence joined:91,1%		Sequence joined:90%	
Methylobacteriaceae	34,7	*Rhodocyclaceae*	9,1	*Methylobacteriaceae*	49	*Methylobacteriaceae*	13	*Cupriavidus*	25	*Methylobacteriaceae*	16,3
Acidovorax defluvii	15,2	*Comamonadaceae*	6,8	*Clostridiales*	7,1	*Rhodocyclaceae*	13	*Methylobacteriaceae*	14	*Streptophyta*	14,1
Corynebacterium kroppenstedtii	13,4	*Actinomycetales*	6,3	*Comamonadaceae*	6,8	*Sphingomonadales*	8,7	*Zoogloea*	12	*Cupriavidus*	10,1
Rhodobacter	6,67	*Syntrophobacter*	6	*Cupriavidus*	6,3	*Sphingomonas*	8	*Exiguobacteraceae*	6,9	*Staphylococcus*	7,94
Corynebacterium	5,04	*Cupriavidus*	5,9	*Corynebacterium*	4,4	*Porphyromonas*	7,6	*Staphylococcus*	6,8	*Faecalibacterium prausnitzii*	7,92
Staphylococcus	3,82	*Sphingomonadales*	5,7	*Sphingomonadales*	3,6	*Comamonadaceae*	7,5	*Streptococcus*	4,3	*Streptococcus*	6,6
Rhodoferax	3,64	*Acidovorax defluvii*	5,6	*Kocuria rhizophila*	3,4	*Cupriavidus*	6,9	*Sphingomonas*	4	*Propionibacterium acnes*	6,13
Cupriavidus	2,94	*Methylobacteriaceae*	5,3	*Acetobacteraceae*	3,1	*ZB2*	5,9	*Streptococcusminor*	2,7	*Corynebacterium*	5,02
Ralstonia	2,58	*Comamonadaceae*	4,8	*Lactobacillus helveticus*	2,4	*Granulicatella*	4,5	*Escherichia coli*	2,6	*Corynebacterium kroppenstedtii*	4,62
Halomonas	2,13	*Streptococcus*	4,3	*Rhodocyclaceae*	1,5	*Propionibacterium acnes*	2,9	*Weeksellaceae*	1,8	*Sphingomonas*	3,86
Comamonadaceae	1,7	*Xenococcaceae*	3,4	*Staphylococcus*	1,4	*Neisseriaceae*	2,8	*Kocuria rhizophila*	1,8	*Enterococcus*	2,83
		Ralstonia	3,3	*Acidovorax defluvii*	1,1	*258ds10*	2,8	*Rhizobiales*	1,5	*Carnobacterium viridans*	1,45
		Bradyrhizobiaceae	3,2			*Arcobacter*	2,6	*Actinomyces*	1,5	*Tatlockia*	1,34
		Chryseobacterium	3,1			*Comamonadaceae*	1,4	*Chitinophagaceae*	1,4	*Sphingomonadales*	1,13
		Ruminococcus	2,7			*Caulobacteraceae*	1,4	*Bradyrhizobiaceae*	1,4		
		Rhodobacter	2,6			*Rhizobiales*	1,3	*Burkholderiales*	1,2		
		Corynebacterium kroppenstedtii	2,6			*Aquabacterium*	1,3	*Parabacteroides distasonis*	1,1		
		Zoogloea	2,1			*Betaproteobacteria*	1				
		Neisseriaceae	1,8								
		Methylobacterium	1,6								
		Rhizobiales	1,6								
		Sphingomonas	1,5								
		Polaromonas	1,5								
		Actinomyces	1,2								

ALS3 MC		ALS3 MD		ALS3 SC		ALS4 MC		ALS4 MD		ALS4 SC	
Number of reads:185149<br>Sequence joined:93,8%		Number of reads:190933<br>Sequence joined:95,6%		Number of reads:174627<br>Sequence joined:95,3%		Number of reads:249140<br>Sequence joined:93,9		Number of reads:172752<br>Sequence joined:95,1%		Number of reads:226642<br>Sequence joined:94,4%	
Methylobacteriaceae	12,2	*Bradyrhizobiaceae*	29	*Methylobacteriaceae*	26	*Cupriavidus*	49	*Methylobacteriaceae*	39	*Methylobacteriaceae*	32,3
Corynebacterium	11,2	*Cupriavidus*	22	*Corynebacterium*	16	*Methylobacteriaceae*	15	*Lachnospira*	18	*Methylophilaceae*	12,8
Acidovorax defluvii	10,5	*Sphingomonadales*	14	*Sphingomonadales*	14	*Ralstonia*	8,8	*Staphylococcus*	9,1	*Zoogloea*	10,5
Rhodocyclaceae	10,1	*Rhodocyclaceae*	7,2	*Cyanobacteria*	9,1	*Rhodocyclaceae*	4,9	*Cupriavidus*	8,3	*Sphingomonas*	7,48
Kocuria rhizophila	6,47	*Staphylococcus*	5,6	*Bradyrhizobiaceae*	8,3	*Corynebacterium kroppenstedtii*	4,6	*Sphingomonadales*	4,1	*Cupriavidus*	6,95
Staphylococcus	5,38	*Methylobacteriaceae*	5,1	*Zoogloea*	6,8	*Corynebacterium*	3,2	*Rhodocyclaceae*	3,9	*Comamonadaceae*	5,15
Corynebacterium kroppenstedtii	5,24	*Haemophilus parainfluenzae*	3,5	*Cupriavidus*	5,3	*Burkholderiales*	2,3	*Aerococcaceae*	3,5	*Streptococcus*	4,95
Comamonadaceae	4,51	*Acidovorax defluvii*	2,1	*Rhodocyclaceae*	3,5	*Actinomycetales*	1,7	*Corynebacterium kroppenstedtii*	2,8	*Sphingomonadales*	4,34
Rhodoferax	4,49	*Afipia*	1,6	*Sphingomonas*	2,4	*Sphingomonadales*	1,5	*Zoogloea*	2,3	*Propionibacterium acnes*	3,8
Neisseriaceae	3,32	*Sphingomonas*	1,5	*Actinomycetales*	1,5	*Anaerococcus*	1,3	*Comamonadaceae*	2,2	*Comamonadaceae*	2,42
Bacillus	3,24	*Kocuria rhizophila*	1,4	*Propionibacterium acnes*	1,2	*Burkholderiaceae*	1,2	*Corynebacterium*	1,5	*Staphylococcus*	1,22
Bradyrhizobiaceae	2,59					*Granulicatella*	1	*Methylobacteriaceae*	0,6	*Betaproteobacteria*	1,19
Propionibacterium acnes	2,31									*Pseudonocardia*	1,09
MIZ46	2,19										
Sphingomonas	2,07										
Sphingomonadales	1,77										
Actinomycetales	1,66										
Cupriavidus	1,48										
Comamonadaceae	1,46										
Rhizobiales	1,04										

ALS5 MC		ALS5 MD		ALS5 SC		ALS6 MC		ALS6 MD		ALS6 SC	
Number of reads:170427<br>Sequence joined:95,2%		Number of reads:239341<br>Sequence joined:91,4%		Number of reads:175021<br>Sequence joined:94%		Number of reads:166514<br>Sequence joined:92,2%		Number of reads:202072<br>Sequence joined:93,8%		Number of reads:238088<br>Sequence joined:94,2%	
Zoogloea	16,7	*Corynebacterium kroppenstedtii*	20	*Zoogloea*	22	*Sphingomonadales*	12	*Methylobacteriaceae*	14	*Methylobacteriaceae*	30,3
Cupriavidus	13,2	*Actinomycetales*	10	*Methylobacteriaceae*	21	*Cupriavidus*	12	*Xanthomonadaceae*	10	*Thermus*	5,18
Bradyrhizobiaceae	12,8	*Cupriavidus*	8,7	*Cupriavidus*	12	*Streptophyta*	12	*OD1*	9,1	*Rhodocyclaceae*	5,01
Methylobacteriaceae	10,8	*Streptococcus*	5,9	*Sphingomonas*	10	*Tepidimonas*	10	*Comamonadaceae*	8,3	*Fusobacterium*	4,95
Kocuria rhizophila	8,78	*Neisseriaceae*	3,9	*Kocuria rhizophila*	9,1	*Methylobacteriaceae*	9,8	*Cupriavidus*	6,4	*Bradyrhizobiaceae*	4,76
Rhodoplanes elegans	7,61	*Staphylococcus*	3,6	*Corynebacterium kroppenstedtii*	8,6	*Chitinophagaceae*	8,1	*Bifidobacterium*	6	*Sphingomonas*	4,47
Sphingomonas	6,7	*Rubellimicrobium*	3,5	*Comamonadaceae*	4,6	*Acidaminobacteraceae*	6	*Weeksellaceae*	4,4	*Comamonadaceae*	4,26
Acidovorax defluvii	5,4	*Nocardioides*	3,2	*Streptococcus*	2,5	*Acidovorax defluvii*	3,5	*Lactobacillaceae*	4	*Sphingomonadales*	4,11
Sphingomonadales	4,88	*Leucobacter*	2,8	*Betaproteobacteria*	1,2	*Roseococcus*	3,4	*Cyanobacteria*	3,5	*Kocuria rhizophila*	3,45
Comamonadaceae	2,59	*Bifidobacterium*	2,8	*Sphingomonas*	1	*Haemophilus parainfluenzae*	2,8	*Bradyrhizobiaceae*	3,4	*Streptophyta*	3,02
Corynebacterium	1,12	*Acidovorax defluvii*	2,7			*Rhodoplanes elegans*	2,3	*Comamonadaceae*	3,3	*Streptococcus*	3,02
Betaproteobacteria	1,08	*Corynebacterium*	2,5			*Propionibacterium acnes*	2,2	*Propionibacterium acnes*	3,3	*Methylobacterium*	2,9
Afipia	1,02	*Bifidobacterium adolescentis*	2,5			*Corynebacterium*	2,2	*Corynebacterium kroppenstedtii*	2,5	*Corynebacterium kroppenstedtii*	2,62
		Haemophilus parainfluenzae	2,5			*Corynebacterium kroppenstedtii*	2,1	*Anaerococcus*	2,2	*Ralstonia*	2,24
		Propionibacterium acnes	2,3			*Comamonadaceae*	1,3	*Hydrogenophilus*	2,1	*Propionibacterium acnes*	2,08
		Comamonadaceae	1,9			*Leptotrichia*	1,1	*Myxococcales*	2,1	*Corynebacterium*	2,08
		Deinococcus	1,9					*Acidovorax defluvii*	1,5	*Actinomycetales*	1,83
		Comamonadaceae	1,8					*Methylobacterium*	1,3	*Coriobacteriaceae*	1,74
		Methylobacteriaceae	1,8					*Rhodocyclaceae*	1,2	*Kineosporiaceae*	1,16
		Finegoldia	1,7							*Granulicatella*	1
		Rhodocyclaceae	1,5								
		Lachnospiraceae	1,5								

ALS7 MS		AL7 MD		ALS7 SC1		ALS7 SC2		ALS 7 SC3	
Number of reads:237300<br>Sequence joined:94,1%		Number of reads:164846<br>Sequence joined:92,9%		Number of reads:192217<br>Sequence joined:91,7%		Number of reads:160991<br>Sequence joined:94,4%		Number of reads:164906<br>Sequence joined:90,2%	
Methylobacteriaceae	34,2	*Cupriavidus*	20	*Streptococcus*	23	*Methylobacteriaceae*	49	*Methylobacteriaceae*	23
Cupriavidus	17,6	*Bacteroides*	14	*Methylobacteriaceae*	15	*Cupriavidus*	14	*Cupriavidus*	14
Staphylococcus	6,2	*Rhodocyclaceae*	9,9	*SR1*	13	*Comamonadaceae*	11	*Sphingomonadales*	10
Acidovorax defluvii	5,34	*Rhodoplanes elegans*	6	*Veillonella dispar*	8,6	*Sphingomonadales*	3,5	*Rhizobiales*	7,2
Corynebacterium kroppenstedtii	4,39	*Streptococcus*	5,5	*Neisseriaceae*	6,8	*Sphingomonas*	3,4	*Bradyrhizobiaceae*	6,7
Sphingomonadales	4,36	*Comamonadaceae*	5,5	*Corynebacterium kroppenstedtii*	6,3	*Capnocytophaga;s__ochracea*	2,4	*Aerococcaceae*	5,5
Haemophilus parainfluenzae	3,73	*Methylobacteriaceae*	4,5	*Ralstonia*	5,1	*Acinetobacter*	1,8	*Propionibacterium acnes*	5,4
Ralstonia	2,43	*Fusobacterium*	3,6	*Methylobacterium*	3,4	*Propionibacterium acnes*	1,8	*Kocuria rhizophila*	3,7
Streptococcus	2,22	*Kocuria rhizophila*	3,5	*Sphingomonadales*	2,1	*Corynebacterium*	1,7	*Staphylococcus*	3,4
Comamonadaceae	1,97	*Haemophilus parainfluenzae*	3,5	*Halomonas*	2,1	*Bradyrhizobiaceae*	1,5	*Caulobacteraceae*	2,7
Corynebacterium	1,77	*Anaerolinea*	3,3	*Rhodocyclaceae*	2,1	*Gemellaceae*	1,1	*Comamonadaceae*	2,6
Acinetobacter	1,58	*Corynebacterium kroppenstedtii*	3,1	*Bradyrhizobiaceae*	2			*Sphingomonas*	2,5
Propionibacterium acnes	1,52	*Acidovorax defluvii*	3,1	*Kocuria rhizophila*	1,1			*Nevskia ramosa*	2,4
Prevotella melaninogenica	1,45	*Aerococcaceae*	1,6	*Rhodococcus*	1			*Corynebacterium kroppenstedtii*	1,4
Kocuria rhizophila	1,41	*Burkholderia*	1,3					*Dermacoccus*	1
		Selenomonas	1,3						
		Propionibacterium acnes	1,2						
		Staphylococcus	1,1						

ALS8 MD		ALS8 SC		ALS9 MC		ALS9 MD		ALS9 SC	
Number of reads:168458<br>Sequence joined:92,2%		Number of reads: 230769<br>Sequence joined:94,1%		Number of reads:151705<br>Sequence joined:89,4%		Number of reads:197724<br>Sequence joined:92,4%		Number of reads:179587<br>Sequence joined:84,6%	
Methylobacteriaceae	18,3	*Methylobacteriaceae*	20	*Methylobacteriaceae*	25	*Acidovorax defluvii*	47	*Bacillus*	29
Cupriavidus	11,8	*Citrobacter*	16	*Fusobacterium*	12	*Methylobacteriaceae*	9,7	*Cupriavidus*	8,2
Comamonadaceae	9,46	*Acinetobacter*	7,9	*Cupriavidus*	9	*Sphingomonadales*	8,6	*Streptococcus*	8,1
Sphingomonadales	7,67	*Kocuria rhizophila*	4,7	*Actinomycetales*	8,9	*Sphingomonas*	6,9	*Methylobacteriaceae*	8,1
Bradyrhizobiaceae	7,39	*Janthinobacterium*	3,5	*Halomonas*	7	*Cupriavidus*	6,1	*Comamonadaceae*	6,8
Staphylococcus	5,47	*Exiguobacteraceae*	3,4	*Afipia*	5,5	*Streptophyta*	3,5	*Staphylococcus*	6,7
Sphingomonas	4,08	*Sphingomonas*	3	*Kingella*	5,2	*Alphaproteobacteria*	3,2	*Bradyrhizobiaceae*	5,2
Haemophilus parainfluenzae	3,69	*Streptophyta*	3	*Bradyrhizobiaceae;g__;s__*	4,1	*Comamonadaceae*	1,6	*Zoogloea*	4,6
Kocuria rhizophila	3,65	*Leucobacter*	2,9	*Leucobacter*	2,7	*MIZ46*	1	*Sphingomonadales*	3,2
Phenylobacterium	2,59	*Bradyrhizobiaceae*	2,9	*Comamonadaceae*	2,1			*Enterococcus*	3,2
Corynebacterium kroppenstedtii	2,54	*Prevotella*	2,4	*Kineosporiaceae*	1,8			*Acidovorax defluvii*	2,4
Propionibacterium acnes	2,3	*Cupriavidus*	2,1	*Halomonas pacifica*	1,6			*Corynebacterium kroppenstedtii*	1,6
Methylobacterium	2,26	*Brachybacterium;s__conglomeratum*	1,7	*Geodermatophilus*	1,3			*Acinetobacter*	1,5
Zoogloea	1,96	*Haemophilus parainfluenzae*	1,5	*Methylobacterium*	1,3			*Corynebacterium*	1,1
Caulobacteraceae	1,84	*Acetobacteraceae*	1,5	*Staphylococcus*	1,3			*Acinetobacterlwoffii*	1
Alloiococcus	1,75	*Staphylococcus*	1,3	*Neisseriaceae*	1				
Arthrobacter	1,64	*Corynebacterium kroppenstedtii*	1,2						
Ralstonia	1,62	*Bacillus*	1,2						
Rhizobiales	1,19	*Sphingomonadales*	1,2						
		ZB2	1,1						

ALS10 MC		ALS10 MD		ALS10 SC		ALS11 MC		ALS11 MD		ALS11 SC	
Number of reads:164795<br>Sequence joined:81,1%		Number of reads:223545<br>Sequence joined:92,5%		Number of reads:181653<br>Sequence joined:92,4%		Number of reads:145602<br>Sequence joined:89,5%		Number of reads:148579<br>Sequence joined:87,9%		Number of reads:234221<br>Sequence joined:92,6%	
Methylobacteriaceae	24	*Streptococcus*	14	*Methylobacteriaceae*	24	*Corynebacterium*	22	*Cupriavidus*	17	*Corynebacterium*	33,5
Zoogloea	16,4	*Bradyrhizobiaceae*	11	*Rhodocyclaceae*	8,8	*Methylobacteriaceae*	7,7	*Phyllobacterium;s__*	15	*Cupriavidus*	9,95
Sphingomonadales	10,7	*Sphingomonadales*	10	*Hymenobacter;s__*	6,4	*Rhizobiales*	7,3	*Kocuria rhizophila*	11	*Facklamia*	8,83
Cupriavidus	9,48	*Cupriavidus*	8,9	*Haemophilus parainfluenzae*	5,8	*Sphingomonadales*	7,1	*Pseudomonas*	8	*Enterococcus*	7,39
Propionibacterium acnes	6,23	*Methylobacteriaceae*	7,1	*OD1*	5,6	*Streptococcus*	7,1	*Zoogloea*	7	*Methylobacteriaceae*	6,17
Corynebacterium	6,19	*Comamonadaceae*	6,4	*Aeromonadaceae*	5,3	*Acidovorax defluvii*	7	*Sphingomonadales*	6,6	*Lactobacillus helveticus*	6,04
Bradyrhizobiaceae	3,93	*Phenylobacterium*	5,4	*Corynebacterium kroppenstedtii*	4,1	*Kocuria rhizophila*	6,1	*Methylobacteriaceae*	5,2	*Corynebacterium;Other*	5,34
Gemellaceae	3,82	*Sphingomonas*	4,6	*Propionibacterium acnes*	4	*Anaerococcus*	4,4	*Sphingomonas*	4,4	*Actinomycetales*	4,22
Acidovorax defluvii	3,29	*Staphylococcus*	3,4	*Staphylococcus*	3,8	*Rhizobiales*	4,2	*ZB2*	3,5	*Bradyrhizobiaceae*	3,38
Brevibacterium	3,27	*Haemophilus parainfluenzae*	3,4	*Acinetobacter*	3,6	*Cupriavidus*	4	*Acidovorax defluvii*	2,8	*Zoogloea*	2
Kingella	2,4	*Anaerococcus*	2,9	*Cupriavidus*	3,4	*Sphingomonas*	3,6	*Rothia dentocariosa*	2,5	*Propionibacterium acnes*	1,77
Staphylococcus	1,82	*Citrobacter*	2,6	*Kingella*	2,9	*Rhodocyclaceae*	3,2	*Staphylococcus*	2,2	*Sphingomonadales*	1,31
Rhodocyclaceae	1,02	*Acidovorax defluvii*	2,5	*Peptoniphilus*	2,9	*Bacillus*	2,7	*Corynebacterium kroppenstedtii*	2,2	*Anaerococcus*	1,31
		Streptococcus	2,4	*Thermus*	2,2	*Prevotella intermedia*	1,7	*Hymenobacter*	1,5	*Staphylococcus*	1,27
		Kingella	1,9	*Streptococcus*	2,1	*Actinomycetales*	1,6	*Propionibacterium acnes*	1,2		
		Rhodoplanes elegans	1,4	*Sphingomonadales*	1,4	*Staphylococcus*	1,5	*S-70*	1,2		
		Streptococcusminor	1,4	*Acinetobacterschindleri*	1,3	*Erythrobacteraceae*	1,3	*Bradyrhizobiaceae*	1,1		
		Propionibacterium acnes	1,3	*Kocuria rhizophila*	1,1	*Corynebacterium kroppenstedtii*	1,3				
				Comamonadaceae	1						

Supplementary Table III. Analysis of the exanucleotide expansion repeat in C9orf72

Patients	Sequence
ALS1 MC	AAGAATAATTTCAGCCCCCACCCCCGCCCGGCAGCAGCATCTT<br>CCTCCCCCTCTTTTACGTTACGCATCCCAGTTTGAGACG**GGGG**<br>**CCGGGGCCGGGGCC**GGGGCTGCGGTTGCGGTGCCTGCGCCC<br>GCGGCGGCGGAGGCGCAGGCGGTGGCGAGTGGGTGAGTGAGG<br>AGGCGGCATCCTGGCGGGTGGCTGTTTGGGGTTCGGCTGCCGG<br>GAAGAGGCGCGGGTAGAAGCGGGGGCTCTCCTCAGAGTCTCG<br>ACGCATTTTTACTTCCCTCTCATTCTCTACCAAGTTGTT
ALS2 MC	TTTTTTCTTTTTCTCTCTTATTTGTGATTTTTTTTTTCCTTTTTTG<br>AGGTATTTTGATACCCCCTTCCCTATACTTACGTTTCGCATCCC<br>AGTTGAGACG**GGGGCCGGGGCCGGGGCC**GGGGCTGCGGTT<br>GCGGTGCCTGCGCCCGCGGCGGCGGAGGCGCAGGCGGTGGCG<br>AGTGGGTGAGTGAGGAGGCGGCATCCTGGCGGGTGGCTGTTT<br>GGGGTTCGGCTGCCGGGAAGAGGCGCGGGTAGAAGCGGGGGC<br>TCTCCTCAGAGCTCTGTACGCATTTTTACTTCCCTCTCATCTCT<br>ACCAAGTTTTT
ALS3 MC	ATACTCATGTCTTCCTTTTTCGTATTAAGAAGCATTATCAGGGT<br>TATTGTCTCAGAGCGGTATTTTTTGTGAACGTTTTATAAATATA<br>AACAAAGGGTGTTCCGCGCACATTTCCCAGAAAAGTCCCGCCT<br>GACGTCGCTAGCTGTACAAAAATCTGGCTTTAAGGAACCAATT<br>CAGTGGACTGGTTCCTGTACCAATGTCGTGCAGGAAGAGGCCC<br>TATTTCCCATGATTCCTTCATATTGGCATATCCTTTCCAGGCCT<br>TTAGAGAGATAGTTAGAATCATTTTGACTGTAATCTTTTGTTTC<br>GCATCCCAGTTGAGACG**GGGGCCGGGGCCGGGGCC**GGGGCT<br>GCGGTTGCGGTGCCTGCGCCCGCGGCGGCGGAGGCGCAGGCG<br>GTGGCGAGTGGGTGAGTGAGGAGGCGGCATCCTGGCGGGTGG<br>CTGTTTGGGGTTCGGCTGCCGGGAAGAGGCGCGGGTAGAAGC<br>GGGGGCTCTCCTCAGAGCTACGACGCATTTTACTTCCGTGCTC<br>ATCTCTACCAGCGTTCAACGGTGG
ALS4 MC	CCTCCTGTGAAAAGCTCGGTCGAGAATGAGAGGGAAGTAAAA<br>ATGCGTCGAGCTCTGAGGAGAGCCCCCGCTTCTACCCGCGCCT<br>CTTCCCGGCAGCCGAACCCCAAACAGCCACCCGCCAGGATGC<br>CGCCTCCTCACTCACCCACTCGCCACCGCCTGCGCCTCCGCCG<br>CCGCGGGCGCAGGCACCGCAACCGCAGCCCC**GGCCCCGGCC**<br>**CCGGCCCC**CGTCTCAAACTGGGATGCGTAAAG
ALS5 MC	CAAAATTACAGCATCCCAGTTTGAGACGGGGGCCGGGGCCGG<br>GGCCGGGGAAGCGAATCGTTAGATACAGTTTACGCATTACCAT<br>ACGCATCACGTTTGAGACG**GGGGCCGGGGCCGGGGCC**GGG<br>GCTGCGGTTGCGGTGCCTGCGCCCGCGGCGGCGGAGGCGCAG<br>GCGGTGGCGAGTGGGTGAGTGAGAGGCGGCATCCTGGCGGGT<br>GGCTGTTTGGGGTTCGGCTGCCGGAAGAGGCGCGGGTAGAAG<br>CGAACTCTCCTCAGAAGCTTCGAACGCATTTTTATTTCCCTTTC<br>ATTTTTGACGGAGGTGGTT
ALS6 MC	TTTACGCATCCCAGTTTGAGACG**GGGGCCGGGGCCGGGGCC**<br>GGGGCTGCGGTTGCGGTGCCTGCGCCCGCGGCGGCGGAGGCG<br>CAGGCGGTGGCGAGTGGGTGAGTGAGGAGGCGGCATCCTGGC<br>GGGTGGCTGTTTGGGGTTCGGCTGCCGGGAAGAGGCGCGGGT<br>AGAAGCGGGGGCTCTCCTCAGAGCTCGACGCATTTTTACTTCC<br>CTCTCATCTCTACCGAGCTGTT
AL7 MC	TTTATTTCTTATTTTTCTTGTTTTTTTTTTATTTTTATTTTTGTTTT<br>TTTACTTTTTTAGAGATATTTAGAAGTAAAGCACCTTAGAATTC<br>GTTTACGCATCCCAGTTTGAGAC**GGGGGCCGGGGCCGGGGC**<br>**C**GGGGCTGCGGTTGCGGTGCCTGCGCCCGCGGCGGCGGAGGC<br>GCAGGCGGTGGCGAGTGGGTGAGTGAGGAGGCGGCATCCTGG<br>CGGGTGGCTGTTTGGGGTTCGGCTGCCGGGAAGAGGCGCGGG<br>TAGAAGCGGGGGCTCTCCTCAGAGTCTACGCACGCATTTTTAC<br>TTCCCTCTCATCTCCGACCAAGGTTGGGT
ALS9 MC	GTGTTCTTATTTTTCTTGTTTTTTTCTTATATGGATATATGTTTT<br>ATGGCTCTTTGAGAGGTATTTGGATTTCTTCCTCCTTTTATTAC<br>GTTACGCATCCCAGTTTGAGACG**GGGGCCGGGGCCGGGGCC**<br>GGGGCTGCGGTTGCGGTGCCTGCGCCCGCGGCGGCGGAGGCG<br>CAGGCGGTGGCGAGTGGGTGAGTGAGGAGGCGGCATCCTGGC<br>GGGTGGCTGTTTGGGGTTCGGCTGCCGGGAAGAGGCGCGGGT

	AGAAGCGGGGGCTCTCCTCAGAGCTCGTACGCATTTTTACTTC<br>CCTCTCAGTCTCTACCAAGACTGTT
ALS10 MC	TCCCAAGATTCCTTCATATGGCATATACGATACAAGGACTTAG<br>GAAAATAATTAGAATAAATTTGACTGTAATTAAGTTTCGCATC<br>CCAGTTTGAGACGGGGGGC**GGGGCCGGGGCC**GGGGCTGCGG<br>TTGCGGTGCCTGCGCCCGCGGCGGCGGAGGCGCAGGCGGTGG<br>CGAGTGGGTGAGTGAGGAGGCGGCATCCTGGCGGGTGGCTGT<br>TTGGGGTTCGGCTGCCGGGAAGAGGCGCGGGTAGAAGCGGGG<br>GCTCTCCTCAGAGTCTCGTACGCTATCTTACGTTACCCTCTCAG<br>CTCGACCCAAAGACGGGT
ALS11 MC	TTATGAGTTTTTCTCAAAAAAGGGAAAGAGTCTCGCGGCTCCT<br>GAAGAGGGAGGGTGAGGAGATGCTTCCTAGAATTACGTTTCG<br>CATTCCAATATGAGACGCGGGCC**GGGGCCGGGGCC**GGGGCT<br>GCGGTTGCGGTGCCTGCGCCCGCGGCGGCGGAGGCGCAGGCG<br>GTGGCGAGTGGGTGAGTGAGGAGGCGGCATCCTGGCGGGTGG<br>CTGTTTGGGGTTCGGCTGCCGGGAAGAGGCGCGGGTAGAAGC<br>GGGGGCTCTCCTCAGATCTACTCTCGCATTTTACTTCCCTGTCG<br>TTCTCGACCGAGGTTGGT
ALS1 MD	CGCTGTTTCGTGGATCTTAACCCTCTGCGCTCTGGTGGCCAATA<br>TATTCCTTCAGAAGCGCCGCGTTTTGTAAGACTTCATGAGGAG<br>TATTCGTCGTATATTTTTGTTCTGCATTTACGTTTCGCATTCCA<br>GTTGAGACG**GGGGCCGGGGCCGGGGCC**GGGGCTGCGGTTGC<br>GGTGCCTGCGCCCGCGGCGGCGGAGGCGCAGGCGGTGGCGAG<br>TGGGTGAGTGAGGAGGCGGCATCCTGGCGGGTGGCTGTTTGG<br>GGTTCGGCTGCCGGGAAGAGGCGCGGGTAGAAGCGGGGGCTC<br>TCCTCAGAGCTCGACGCATTTTTACTTCCCTCTCATCTCGACCG<br>AGGCGGG
ALS2 MD	GCCTTCTACATCATTATTATTTGGAATGAATACTTGTATTGTTT<br>GCAGATTCATGGGTGATTCGTTACAAGGGTTTGTATCACACCC<br>TGAATCTTAGCTTATTAGACCAAACACGAATTCCTAAGTGTTC<br>TTACCACCTCATCTTTCGCATATCATTTACAAAAGATTTACGTT<br>ACGCACCCCGTTTGAGACG**GGGGCCGGGGCCGGGGCC**GGG<br>GCTGCGGTTGCGGTGCCTGCGCCCGCGGCGGCGGAGGCGCAG<br>GCGGTGGCGAGTGGGTGAGTGAGGAGGCGGCATCCTGGCGGG<br>TGGCTGTTTGGGGTTCGGCTGCCGGGAAGAGGCGCGGGTAGA<br>AGCGGGGCTCTCCTCAGAGCTCGACGCATTTTTACTTTCCCTCT<br>CTTCTTACGAAGCGGT
ALS3 MD	CAAGGCAGCATATTAACTCAGACATTACAAAAGTTACCAAGA<br>CCTGCTTACCATTTACCCAAATCTATGGTATCCTTATATTTTAC<br>ATTACGCATCCCAGTTTGAGACG**GGGGCCGGGGCCGGGGCC**<br>GGGGCTGCGGTTGCGGTGCCTGCGCCCGCGGCGGCGGAGGCG<br>CAGGCGGTGGCGAGTGGGTGAGTGAGGAGGCGGCATCCTGGC<br>GGGTGGCTGTTTGGGGTTCGGCTGCCGGGAAGAGGCGCGGGT<br>AGAAGCGGGGGCTCTCCTCAGAGCTCGACGCATTTTTACTTCC<br>CTCTCATTCTCGACCGAAGATGTT
ALS4 MD	CACAATATTAACTCAAACCTTAGAAAAAGCACCAAAACCAGC<br>AAAAAAAAAAAAAAACCCAACCTTTTTTACGCTTACGTTACGC<br>ATCCCCATTTGAGACG**GGGGCCGGGGCCGGGGCC**GGGGCTG<br>CGGTTGCGGTGCCTGCGCCCGCGGCGGCGGAGGCGCAGGCGG<br>TGGCGAGTGGGTGAGTGAGGAGGCGGCATCCTGGCGGGTGGC<br>TGTTTGGGGTTCGGCTGCCGGGAAGAGGCGCGGGTAGAAGCG<br>GGGGCTCTCCTCAGAGCTGACGCATTTTTACTTCCCTCTCATCT<br>CGATCGA
ALS5 MD	TTGTTTCGGCACCAAAATCAACGGGACTTTCCAATAATGTCGT<br>AATTACGCATCCCAGTTTGAGACGGAGGCCGGGGCCGGGGCC<br>GGGGCGCCCGCAGACCCAAGCGGGTTTACGTTACGCACCCCAT<br>TTGAGACG**GGGGCCGGGGCCGGGGCC**GGGGCTGCGGTTGCG<br>GTGCCTGCGCCCGCGGCGGCGGAGGCGCAGGCGGTGGCGAGT<br>GGGTGAGTGAGGAGGCGGCATCCTGGCGGGTGGCTGTTTGGG<br>GTTCGGCTGCCGGGAAGAGGCGCGGGTAGGAAGCGGGGGCTC<br>TCCTCAGGAGCTCGGACGCATTTTTACTTCCTTCTCATTCTCGA<br>CGGATGCTGGT
ALS6 MD	ATATATTAACATTTTCACTTTGTACCTGCTTTTCTTTTATCATTG<br>ATCAATGGTTTTGGGTTTTAGTGTATTTTTACGTTTTGCATTAC<br>TTTACGCATCCCCATTTGAGACG**GGGGCCGGGGCCGGGGCC**<br>GGGGCTGCGGTTGCGGTGCCTGCGCCCGCGGCGGCGGAGGCG<br>CAGGCGGTGGCGAGTGGGTGAGTGAGGAGGCGGCATCCTGGC

	GGGTGGCTGTTTGGGGTTCGGCTGCCGGGAAGAGGCGCGGGT<br>AGAAGCGGGGGCTCTCCTCAGAGCTCGACGCATTTTTACTTCC<br>CTCTCATCTCTACGAGGCTGT
ALS7 MD	GGCACAAAGATGAACGGGACTTTCCAAAATGTCGTAGAAATT<br>CCACCCCATCGACGCAAGTAGACGGTAGGGTGAGTTCGCGGT<br>AGGTCTAGATAAGCAGAACTCCTTTCAAGTACGGTCAGATCGC<br>CGGCAGAGACCATAAACGCCCGGCTGGCTTCCATCGAAGATTC<br>TAGATTAGAGGAACAAGTTACAACATTTAGGCTTGCTTGGAGG<br>AGTTCGAACCATGAATGCGATTCTGATTTGGAGGTCAGGAGTG<br>AGCGAGGATAACTACACATTCACGGGAATTGATAAGTTATCAC<br>CACATGAACGTCAAATGACAGAATTTTAAGTCAGAAATTAGG<br>AATGTCACCCAGACGTGCTTCCCATTCAGCCCACTTACGCATC<br>CCAGTTGTCAGTTACGCATCCCGTTTGATTACGGGGCCCGTCC<br>**GGGGCCGGGCCGGGGCC**GGCGCCGCCTGCGCCCACGGCGGG<br>GGAGACGCAGGCGGCGGCGAGGGTGTGAGTGAGGTTGCGGCA<br>GCCTGGCGGGTGGATGTTTTGGGTTCGGCTGCCGGGAAGAGGC<br>GCGGGCGAAGCGGGGGCTCTCCTCAGAGCTGGAGCGCATCCT<br>ACATTACATACTCATCTTATACCGGGGTGGTTTA
ALS8 MD	TTTACGCATCCCATTACGCATCCCAGTTTGAGACG**GGGGCCG**<br>**GGGCCGGGGCC**GGGGCTGCGGTTGCGGTGCCTGTGCCTGCGG<br>CGGCGGAGGCGCAGGCGGTGGCGAGTGGGTGAGTGAGGAGGC<br>GGCATCCTGGCGGGTGGCTGTTTGGGGTTCGGCTGCCGGGAAG<br>AGGCGCGGGTAGAAGCGGGGGCTCTCCTCAGAGCTCGACGCA<br>TTGTTACTTCCCTCTCAGTCTCGACCGATGCCCTGA
ALS9 MD	CCATTACGCATCCCCATTTGAGACG**GGGGCCGGGGCCGGGG**<br>**CC**GGGGCTGCGGTTGCGGTGCCTGCGCCCGCGGCGGCGGAGG<br>CGCAGGCGGTGGCGAGTGGGTGAGTGAGGAGGCGGCATCCTG<br>GCGGGTGGCTGTTTGGGGTTCGGCTGCCGGGAAGAGGCGCGG<br>GTAGAAGCGGGGGCTCTCCTCAGAGCTCGACGCATTTTTACTT<br>CCCTCTCATCTCTACCGAGCTGTT
ALS10 MD	TCTTGTGGCTGCCTCTAGACTTTCCGAGCCCTTCGCGAGTGGA<br>GGGCGTCTCTTCCCTTTCGGCGAGTGCGTCTCTCAGCCTCGGTG<br>GGGGGTCGGTTTTACGTTTCCCATTTCGAGAC**GGGGCC**CGGG<br>CCCGGGCCCGGGCCGGGGCTGCGGTTGCGGTGCCTGCGCCCGC<br>GGCGGCGGAGGCGCAGGCGGTGGCGAGTGGGTGAGTGAGGA<br>GGCGGCATCCTGGCGGGTGGCTGTTTGGGGTTCGGCTGCCGGG<br>AAGAGGCGCGGGTAGAAGCGGGGGCTCTCCTCAGAGCTCGAC<br>GCATTTTTACTTCCCTCTCATCTCTACCGAGCGTTCAACGGGAG<br>G
ALS11 MD	ATCAACGAGACCAAAAAAACCACCTCTCCCTCCCCCCCACCCA<br>CCAAATTGGGTTTTACGTTACGCATCCCAGTTTGAGACG**GGGG**<br>**CCGGGGCCGGGGCC**GGGGCTGCGGTTGCGGTGCCTGCGCCC<br>GCGGCGGCGGAGGCGCAGGCGGTGGCGAGTGGGTGAGTGAGG<br>AGGCGGCATCCTGGCGGGTGGCTGTTTGGGGTTCGGCTGCCGG<br>GAAGAGGCGCGGGTAGAAGCGGGGGCTCTCCTCAGAGCTCGA<br>CGCATTTTTACTTCCCTCTCATCTCGACGAAGTTGT
